# Antiphospholipid antibodies detected by line immunoassay differentiate among patients with antiphospholipid syndrome, with infections and asymptomatic carriers

**DOI:** 10.1186/s13075-016-1018-x

**Published:** 2016-05-21

**Authors:** Dirk Roggenbuck, Maria Orietta Borghi, Valentina Somma, Thomas Büttner, Peter Schierack, Katja Hanack, Claudia Grossi, Caterina Bodio, Paolo Macor, Philipp von Landenberg, Francesco Boccellato, Michael Mahler, Pier Luigi Meroni

**Affiliations:** Institute of Biotechnology, Faculty 2, Brandenburg University of Technology Cottbus-Senftenberg, Großenhainer Str. 57, 01968 Senftenberg, Germany; Research and Development Department, Medipan GmbH, Dahlewitz/Berlin, Germany; Department of Clinical Science and Community Health, University of Milan, Milan, Italy; Istituto Auxologico Italiano, Laboratory of Immunorheumatology, Cusano Milanino, Italy; Research and Development Department, GA Generic Assays GmbH, Dahlewitz/Berlin, Germany; Chair Immuntechnology, Department of Biochemistry and Biology, University of Potsdam, Potsdam, Germany; Department of Life Sciences, University of Trieste, Trieste, Italy; Labor Nordwest, Schüttorf, Germany; Max Planck Institute for Infection Biology, Berlin, Germany; Inova Diagnostics, San Diego, CA USA

**Keywords:** Antiphospholipid syndrome, Antiphospholipid antibody, Phospholipid binding proteins, Beta2 - glycoprotein I, Line immunoassay

## Abstract

**Background:**

Antiphospholipid antibodies (aPL) can be detected in asymptomatic carriers and infectious patients. The aim was to investigate whether a novel line immunoassay (LIA) differentiates between antiphospholipid syndrome (APS) and asymptomatic aPL+ carriers or patients with infectious diseases (infectious diseases controls (IDC)).

**Methods:**

Sixty-one patients with APS (56 primary, 22/56 with obstetric events only, and 5 secondary), 146 controls including 24 aPL+ asymptomatic carriers and 73 IDC were tested on a novel hydrophobic solid phase coated with cardiolipin (CL), phosphatic acid, phosphatidylcholine, phosphatidylethanolamine, phosphatidylglycerol, phosphatidylinositol, phosphatidylserine, beta2-glycoprotein I (β2GPI), prothrombin, and annexin V. Samples were also tested by anti-CL and anti-β2GPI ELISAs and for lupus anticoagulant activity. Human monoclonal antibodies (humoAbs) against human β2GPI or PL alone were tested on the same LIA substrates in the absence or presence of human serum, purified human β2GPI or after CL-micelle absorption.

**Results:**

Comparison of LIA with the aPL-classification assays revealed good agreement for IgG/IgM aß2GPI and aCL. Anti-CL and anti-ß2GPI IgG/IgM reactivity assessed by LIA was significantly higher in patients with APS versus healthy controls and IDCs, as detected by ELISA. IgG binding to CL and ß2GPI in the LIA was significantly lower in aPL+ carriers and Venereal Disease Research Laboratory test (VDRL) + samples than in patients with APS. HumoAb against domain 1 recognized β2GPI bound to the LIA-matrix and in anionic phospholipid (PL) complexes. Absorption with CL micelles abolished the reactivity of a PL-specific humoAb but did not affect the binding of anti-β2GPI humoAbs.

**Conclusions:**

The LIA and ELISA have good agreement in detecting aPL in APS, but the LIA differentiates patients with APS from infectious patients and asymptomatic carriers, likely through the exposure of domain 1.

**Electronic supplementary material:**

The online version of this article (doi:10.1186/s13075-016-1018-x) contains supplementary material, which is available to authorized users.

## Background

Antiphospholipid syndrome (APS) represents a chronic disabling systemic autoimmune disorder affecting approximately 1 % of the general population and occurring as the primary disorder or being associated with other systemic autoimmune rheumatic diseases. Clinical manifestations are represented by recurrent arterial/venous thrombosis and/or pregnancy morbidity in the persistent presence of antiphospholipid antibodies (aPL) [1–4].

The current international consensus for the classification of APS values clinical and laboratory criteria equally for the diagnosis of APS [2]. The latter criterion comprises the detection of persistent aPL by solid-phase assays, i.e., IgG and IgM to beta2 - glycoprotein I (β2GPI) and the cardiolipin (CL)-β2GPI complex, and by a functional clotting test, i.e., the lupus anticoagulant (LA).

Appropriate aPL analysis, however, still remains a laboratory challenge due to the heterogeneity of aPL and standardization issues for the required ELISA and clotting tests [5–8]. Recent studies suggested that the epitope specificity of anti-β2GPI antibodies may differentiate between anti-domain (D)1 antibodies that are associated with the manifestations of the syndrome and the anti-D4/5 antibodies, which are not [9]. Apart from β2GPI, other phospholipid (PL)-binding proteins such as prothrombin (PT), annexin V (AnV), and high-molecular weight kininogen have been described [10–12]. Furthermore, the relevance of aPL assay techniques involving the interaction of PL-binding proteins like β2GPI and PT with PLs other than CL, such as phosphatidylserine (PS), is still a matter of debate and not yet included in the classification criteria [13].

For risk stratification in patients with APS, different profiles comprising single, double, and triple positivity of aPL are analyzed. Triple positivity in particular seems to be associated with a higher risk for the appearance of clinical APS manifestations [14]. In this context, LA positivity seems to be the best predictor, whereas medium/high levels of IgG to CL and β2GPI are more indicative than low levels thereof and IgM, although it has recently been suggested that the IgM isotype also has predictive value [14, 15].

There is growing evidence that aPL are pathogenic, although aPL alone are not sufficient to induce APS, and probably perpetuate APS. A “second hit” is required to support these pathophysiological processes [16]. Factors such as traditional cardiovascular risks (e.g., hypertension, diabetes mellitus, and obesity), acquired thrombotic risks (e.g., smoking, oral contraception, and pregnancy), genetic factors in hypercoagulation (e.g., factor V Leiden or II mutation, deficiency of ATIII, and protein C and S), and probably most important infections can provide the required triggers for a second hit. It is still unknown whether the persistent presence of aPL in asymptomatic carriers means that they have not yet met the right second hit or whether their aPL do not display pathogenic activity. Up to date, however, current techniques included in the classification criteria have not allowed differentiation of aPL in patients with APS and those in asymptomatic aPL-positive (+) carriers.

Novel assay techniques have been proposed for aPL testing, such as chemiluminescence-based methods or fluorescence enzyme immunoassays. Recently, a new technique employing a hydrophobic solid phase for the simultaneous detection of different aPL has been developed [13, 17–20]. Remarkably, aPL detected by such line immunoassays (LIAs) appear to be more closely associated with the APS phenotype than those detected by enzyme-linked immunosorbent assay (ELISA) [18, 21]. We speculated on whether LIAs demonstrating improved performance characteristics in comparison with ELISA can be the first test able to detect aPL assisting in the differentiation of patients with APS from asymptomatic aPL+ carriers and aPL-positive patients with infectious diseases.

## Methods

### Patients and controls

In total, 207 individuals were enrolled into the study, comprising 61 patients with APS diagnosed in accordance with the international APS classification criteria and 146 controls (Table 1). Patients with APS were further classified as having primary APS (PAPS) with arterial and/or venous thrombosis in the absence of any other related disease, obstetric APS (OAPS) with pregnancy-related complications listed in the classification criteria (early pregnancy loss, intrauterine death, premature birth, pre/eclampsia, and intrauterine growth retardation), and secondary APS (SAPS) in which the syndrome occurs alongside another autoimmune disease [2]. Further, we included 24 aPL+ individuals with no clinical APS manifestations during at least 3 years of follow up (aPL+ asymptomatic carriers). As disease controls we included 73 patients suffering from infectious diseases (infectious diseases controls (IDC)): 3 of these patients were infected with Epstein-Barr virus, 14 with *Toxoplasma gondii*, 24 with cytomegalovirus (CMV), 8 with Rubella virus, 1 with hepatitis C and 23 with *Treponema pallidum* displaying a positive Venereal Disease Research Laboratory test result (VDRL+)). All the patients were attending the outpatient clinic at the Division of Rheumatology of the University of Milan. The study was approved by the local ethical committee (Comitato Etico Milano Area B; 08.07.2014, CS-GA-115565) and complies with the World Medical Association Declaration of Helsinki on the ethical conduct of research involving human subjects and/or animals. Written informed consent was obtained from each patient. All sera had been stored at –20 °C.

**Table 1:** Characteristics of 61 patients with antiphospholipid syndrome and 146 controls enrolled in the study

	Number	Median age, years	Age range, years	Gender f/m
PAPS^*^	34	46	21–75	23/11
arterial thrombosis	23	47	21–66	15/8
venous thrombosis	12	42	28–63	8/4
thrombotic and obstetric manifestations	5	41	33–51	5/0
OAPS^a^	22	39	27–62	22/0
early pregnancy loss (<10th week of gestation)	11	38	34–45	11/0
intrauterine death (>10th week of gestation)	14	40	27–57	14/0
premature birth	5	37	33–42	5/0
fetuses with intrauterine growth retardation	9	35	27–42	9/0
pre/eclampsia	6	34	27–37	6/0
SAPS^b^	5	38	24–58	4/1
aPL+	24	42	22–71	22/2
IDC	50	35	6–86	46/4
VDRL+	23	36	19–58	2/21
HS	49	37	19–68	9/40

*PAPS* primary antiphospholipid syndrome, *OAPS* obstetric primary antiphospholipid syndrome, *HS* healthy subjects, *aPL+*, anti-phospholipid antibody positive, *IDC* infectious diseases controls, *VDRL+* Venereal Disease Research Laboratory test positive, *f* female, *m* male. ^a^Patients with PAPS or OAPS may have more than one of the indicated clinical manifestations. ^b^Secondary antiphospholipid syndrome (*SAPS*): three were associated with systemic lupus erythematosus, two out of three with arterial thrombosis and one with arterial thrombosis and intrauterine death; two were associated with undifferentiated connective tissue disease and with intrauterine deaths

Sera with APS characterized for reactivity against domain 1 (D1) or domains 4/5 (D4/5) were also included. The domain specificity was carried out by solid-phase assays as previously described [22]. In detail, five patients with APS, who were negative for anti-D1 and positive for anti-D4/5 antibodies, and 9 patients with APS who were positive for anti-D1 and negative for anti-D4/5 antibodies, were analyzed for their reactivity by LIA. The clinical and laboratory features of these patients are reported in Additional file 1: Table S1.

### Monoclonal and polyclonal antibodies against PL-binding proteins

To investigate the interaction with β2GPI in the novel assay environment, we employed the chimeric human monoclonal IgG (humoAb) HCAL, composed of human k and γ constant regions and variable regions from the mouse monoclonal β2GPI‐dependent anti-CL antibody (aCL) WBCAL‐1 [23]. The humoAb HCAL was from Inova Diagnostics (San Diego, CA, USA). To determine β2GPI domain reactivity we tested MBB2 - a human minibody containing a single chain fragment variable fused to an IgG1 CH2-CH3-domain that recognizes D1 of human ß2GPI [24]. The humoAb RR7F interacting with PL in the ELISA was used to analyze the reactivity to PL immobilized on the hydrophobic membrane employed in LIA [25].

An anti-PT (aPT) moAb (Kerafast, Boston, USA) and an anti-AnV (aAnV) (Cusabio, Wuhan, China) polyclonal antibody were employed to investigate the binding to PT and AnV. To reveal their specific binding, polyclonal anti-mouse and anti-rabbit IgG labeled with peroxidase were used as secondary antibodies, respectively.

### Interaction of PL-binding proteins with PL

The PL-binding proteins, human β2GPI purified from pooled plasma as described elsewhere [26], purified human PT (Arotec Diagnostics, Wellington, New Zeeland), and recombinant human AnV (Diarect, Freiburg, Germany) were used. These PL-binding proteins were investigated for their binding to PL immobilized on the LIA membrane. Ultrapure Bovine Serum Albumin (BSA; Sigma, St Louis, MI, USA), which was free of any β2GPI contamination was used in some experiments.

### Inhibition of aPL reactivity by CL micelles

For aPL inhibition experiments, CL micelles prepared in a suspension as described elsewhere were employed [27]. Briefly, aß2GPI and aPL humoAbs at a dilution giving positive results in the LIA (1.5 times the cutoff of 50 optical density units) were incubated with 10 mg/L CL micelles in PBS for 1 h at 37 °C on a rotator and subsequently overnight at 4 °C. After ultracentrifugation at 16,000 rpm for 45 minutes, the supernatant was collected and the remaining aß2GPI and aPL reactivity determined in the LIA.

### ELISA for the detection of aCL and aß2GPI

For the detection of aCL and aß2GPI in patient sera, commercially available solid-phase ELISAs employing purified human ß2GPI in complex with CL and human ß2GPI were used, respectively (GA Generic Assays GmbH, Dahlewitz, Germany). Sera were considered positive when their concentration exceeded the cutoff of 10 U/mL for IgG and IgM, respectively. All samples have been tested by respective in-house assays as described elsewhere [22]. The results for the two techniques were comparable (data not shown).

### LIA for the detection of aPL

Antibodies against CL, phosphatidic acid (aPA), phosphatidylcholine (aPC), phosphatidylethanolamine (aPE), phosphatidylglycerol (aPG), phosphatidylinositol (aPI), PS (aPS) and the PL-binding proteins ß2GPI, AnV, and PT were detected in patient sera simultaneously using a commercially available LIA in accordance with the recommendations of the manufacturer (GA) [17]. Briefly, CL, PA, PC, PE, PG, PI, PS, and ß2GPI, AnV and PT were sprayed onto a polyvinylidene difluoride (PVDF) membrane in lines for immobilization as described for glycolipids [28]. A mixture of human IgG and IgM was immobilized likewise as the reaction control band.

Serum samples were diluted 1 in 33 (30 μL + 1 mL) and incubated for 30 minutes at room temperature while shaking to allow sufficient binding of autoantibodies to the PL and proteins immobilized on the PVDF membrane. Unbound serum components were removed by the following wash step with 1 mL wash buffer containing 10 mM TRIS with 0.1 % Tween 20 for 5 minutes. In a further incubation step of 15 minutes at room temperature, the bound autoantibodies reacted specifically with anti-human IgG or IgM conjugated to horseradish peroxidase (POD). Excessive conjugate was separated from the solid-phase immune complexes by an additional wash step. After addition of 50 μL precipitating tetramethylbenzidine as substrate for staining, stripes were dried for at least 30 minutes at room temperature.

Processed strips were analyzed densitometrically employing a scanner with the evaluation software Dr. DotLine Analyzer (GA Generic Assays). Optical density values equaling or above 50 were scored positive. This cutoff was determined by calculating the 99 % percentile of 150 apparently healthy individuals as recommended by the international classification criteria for aPL testing and Clinical and Laboratory Standards Institute (CLSI) guideline C28-A3 [2, 29]. Linearity of dilution with optical density values was demonstrated in the range from 10 to 80 optical density units for most of the aPL-positive samples.

### LA testing

Analysis of LA was performed in accordance with the international recommendations as described recently [30]. LA activity was evaluated in citrated plasma using the ACL TOP coagulation system (Instrumentation Laboratory SpA, Milan, Italy) by the HemosIL™ Silica Clotting Time (SCT) and diluted Russell’s Viper Venon (dRVVT) screen/confirm assays, together with dRVVT and APTT mixing test, according to manufacturer’s protocol and the international guidelines for LA measurement [30].

### Statistical analysis and determination of assay performance characteristics

Fisher’s exact test with two-tailed probability was used to test the differences between groups. Inter-rater agreement statistics were applied for comparison of classifications. Rank correlation of variables was performed by Spearman’s correlation analysis. Medcalc statistical software (Medcalc, Mariakerke, Belgium) was used for all statistical calculations. *P* values <0.05 were considered significant.

## Results

### Reactivity patterns of monoclonal aPL to PL-binding proteins in the LIA

**Fig. 1:**
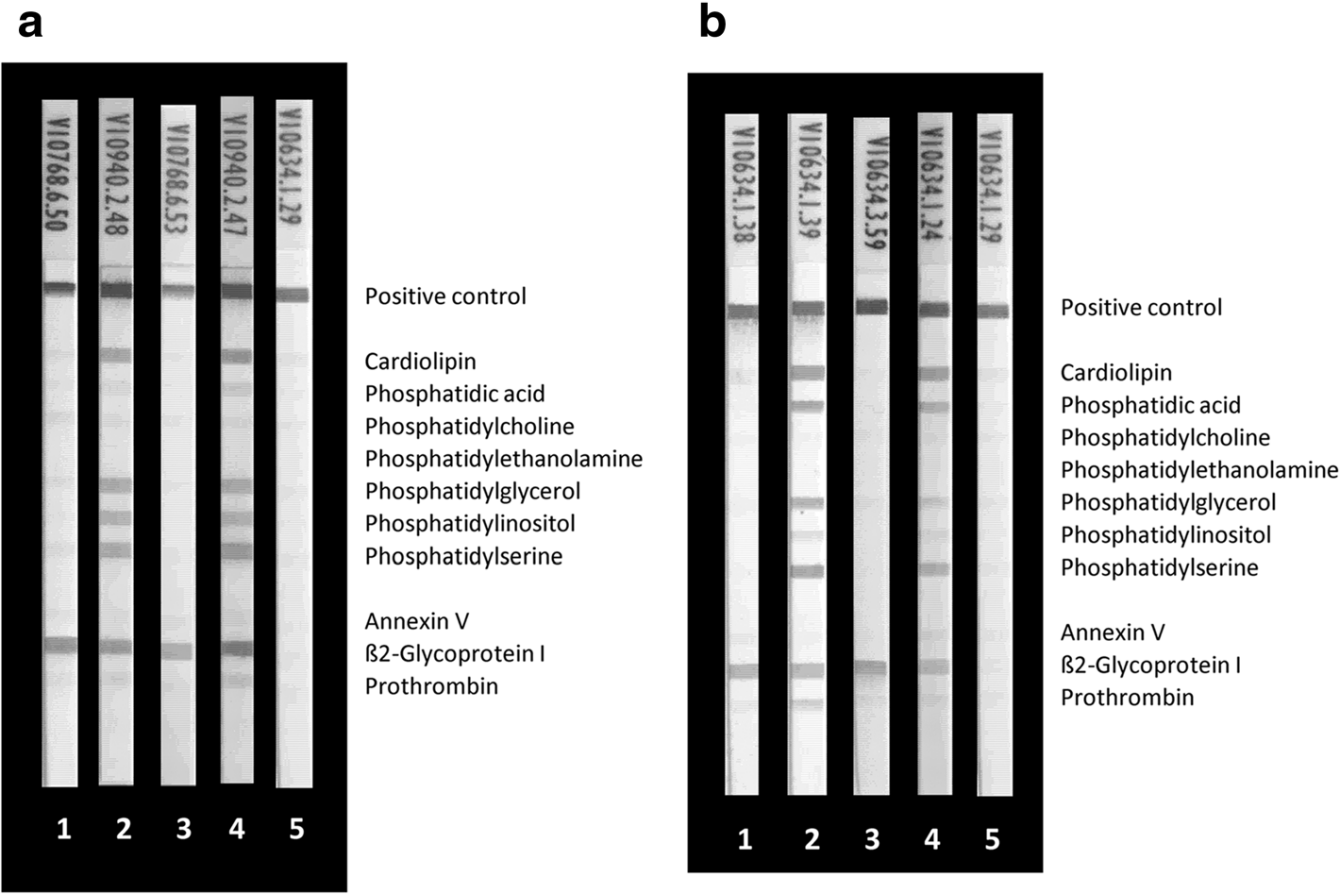
Reactivity of human monoclonal anti-beta2 glycoprotein I (aß2GPI) antibodies MBB2 (**a**) and HCAL (**b**) with phospholipids (PL) and PL-binding proteins by line immunoassay (LIA): MBB2 (0.1 mg/L) and HCAL (0.02 mg/L) were run in the LIA alone or together with serum, ß2GPI, blood donor serum, and bovine serum albumin (BSA). As the positive reaction control a mixture of human IgG and IgM was immobilized. **a** *1*, 0.1 mg/L aß2GPI MBB2; *2*, 0.1 mg/L aß2GPI MBB2 + 10 mg/L ß2GPI; *3*, 0.1 mg/L aß2GPI MBB2 + 10 mg/L BSA; *4*, 0.1 mg/L aß2GPI MBB2 + 30 μL serum (1/33); and *5*, 30 μL serum (1/33). **b** *1*, 0.02 mg/L aß2GPI HCAL; *2*, 0.02 mg/L aß2GPI HCAL + 10 mg/L ß2GPI; *3*, 0.02 mg/L aß2GPI HCAL + 10 mg/L BSA; *4*, 0.02 mg/L aß2GPI HCAL + 30 μL serum (1/33); and *5*, 30 μL serum (1/33)

**Fig. 2:**
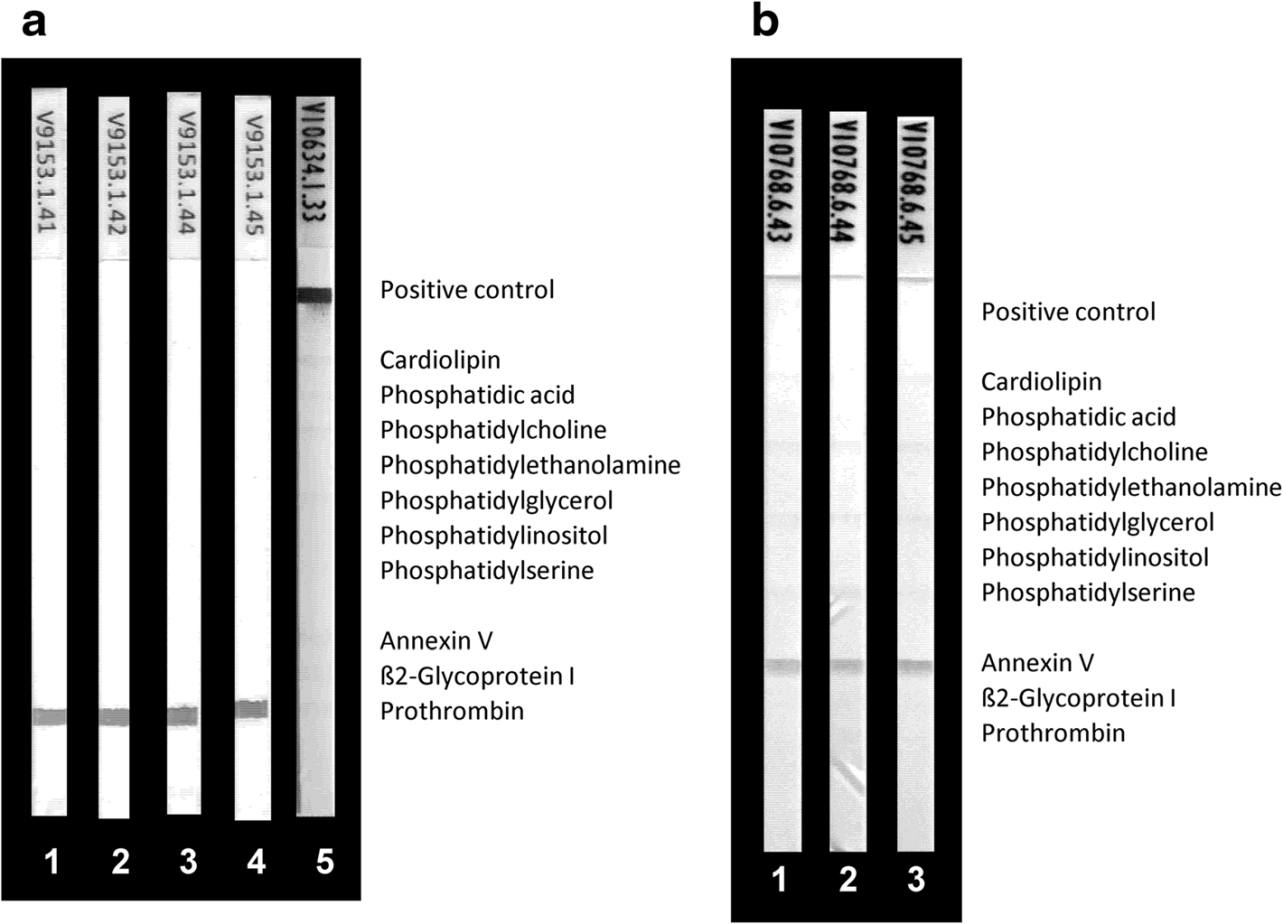
Reactivity of anti-prothrombin (aPT) mouse monoclonal (**a**) and anti-annexin V (aAnV) rabbit polyclonal antibodies (**b**) with phospholipids (PL) and PL-binding proteins by line immunoassay (LIA): aPT and aAnV were run in the LIA alone or together with blood donor serum, bovine serum albumin (BSA) and PT and AnV, respectively. As the positive reaction control a mixture of human IgG and IgM was immobilized. The control band was only revealed when anti-human IgG coupled to horseradish peroxidase was used as secondary antibody conjugate. **a** *1*, 1.0 mg/L aPT; *2*, 1.0 mg/L aPT + 10 mg/L PT; *3*, 1.0 mg/L aPT + 10 mg/L BSA; *4*, 1.0 mg/L aPT + 30 μL serum (1/33); and *5*, 30 μL serum (1/33). **b** *1*, 5.0 mg/L aAnV; *2*, 5.0 mg/L aAnV + 10 mg/L AnV; and *3*, 5.0 mg/L aAnV + 10 mg/L BSA

To analyze the reactivity to PL-binding proteins employed in the LIA, aß2GPI humoAbs MBB2 and HCAL or monoclonal aPT or polyclonal aAnV were tested: (1) alone, (2) with the addition of the respective antigens, (3) with the addition of ultra-pure BSA as a control protein, and (4) with the addition of normal human serum as a source of the PL-binding proteins. As expected, MBB2 and HCAL humoAbs alone reacted only with immobilized ß2GPI on the LIA membrane (Fig. 1). Likewise, monoclonal aPT and polyclonal aAnV also bound specifically to their corresponding immobilized antigens (Fig. 2).

Co-incubation of the aß2GPI humoAbs with serum revealed additional positive bands, indicating reactivity with immobilized CL, PA, PS, and to a lesser extent with PG and PI. In contrast, such additional bands were not detected for the simultaneous incubation of serum with the polyclonal aPT and aAnV (Fig. 2). The additional bands detected for the aß2GPI humoAb incubation with serum could be reproduced by incubating the immobilized PL on the strips either prior to or simultaneously with purified ß2GPI (Fig. 1). ß2GPI interacted with the immobilized negatively charged CL, PA, PS, PG and PI in a dose-dependent manner and was subsequently recognized by the aß2GPI humoAbs (data not shown). Thus, the lower reactivity of the HCAL when incubated with serum is probably due to the lower final concentration of ß2GPI in the diluted serum.

In contrast, incubation of PT with the LIA strips followed by aPT mAb and of AnV followed by polyclonal aAnV did not reveal additional bands, indicating no binding of PT and AnV with the immobilized PL (Fig. 2). High Ca^2+^ ion concentration is known to favor the immunogenic conformational change of PT in solid phase assays [13]; however, the addition of Ca^2+^ ions up to a concentration of 20 mM/L did not affect this reactivity pattern.

**Fig. 3:**
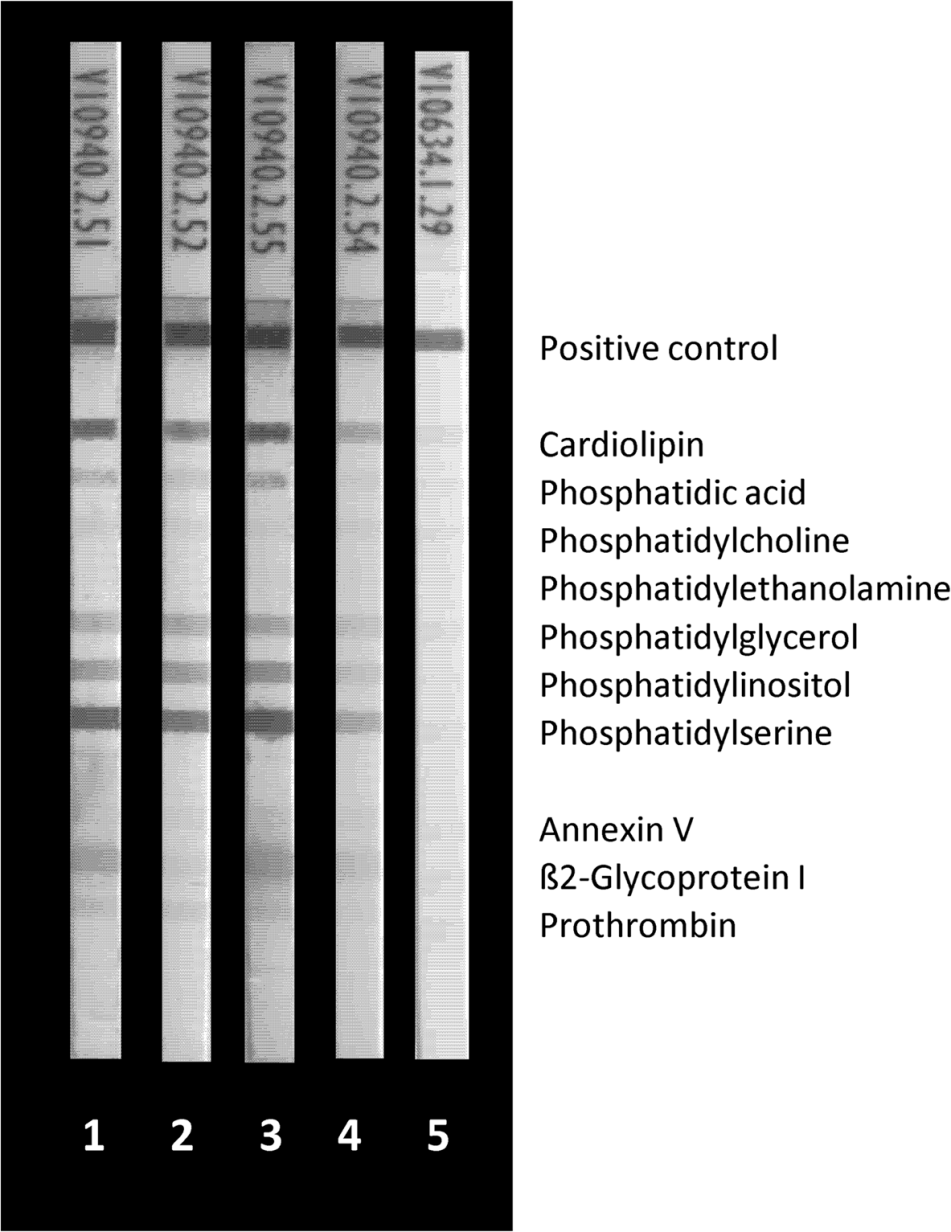
Reactivity of antiphospholipid (aPL) human monoclonal antibody RR7F with PL and PL-binding proteins by line immunoassay (LIA): RR7F (10.0 mg/L) was run in the LIA alone or together with serum, beta2 glycoprotein I (aß2GPI), blood donor serum, and bovine serum albumin (BSA). As positive reaction control a mixture of human IgG and IgM was immobilized. *1*, 10.0 mg/L RR7F; *2*, 10.0 mg/L RR7F + 10 mg/L ß2GPI; *3*, 10.0 mg/L RR7F + 10 mg/L BSA; *4*, 10.0 mg/L RR7F + 30 μL serum (1/33); and *5*, 30 μL serum (1/33)

**Fig. 4:**
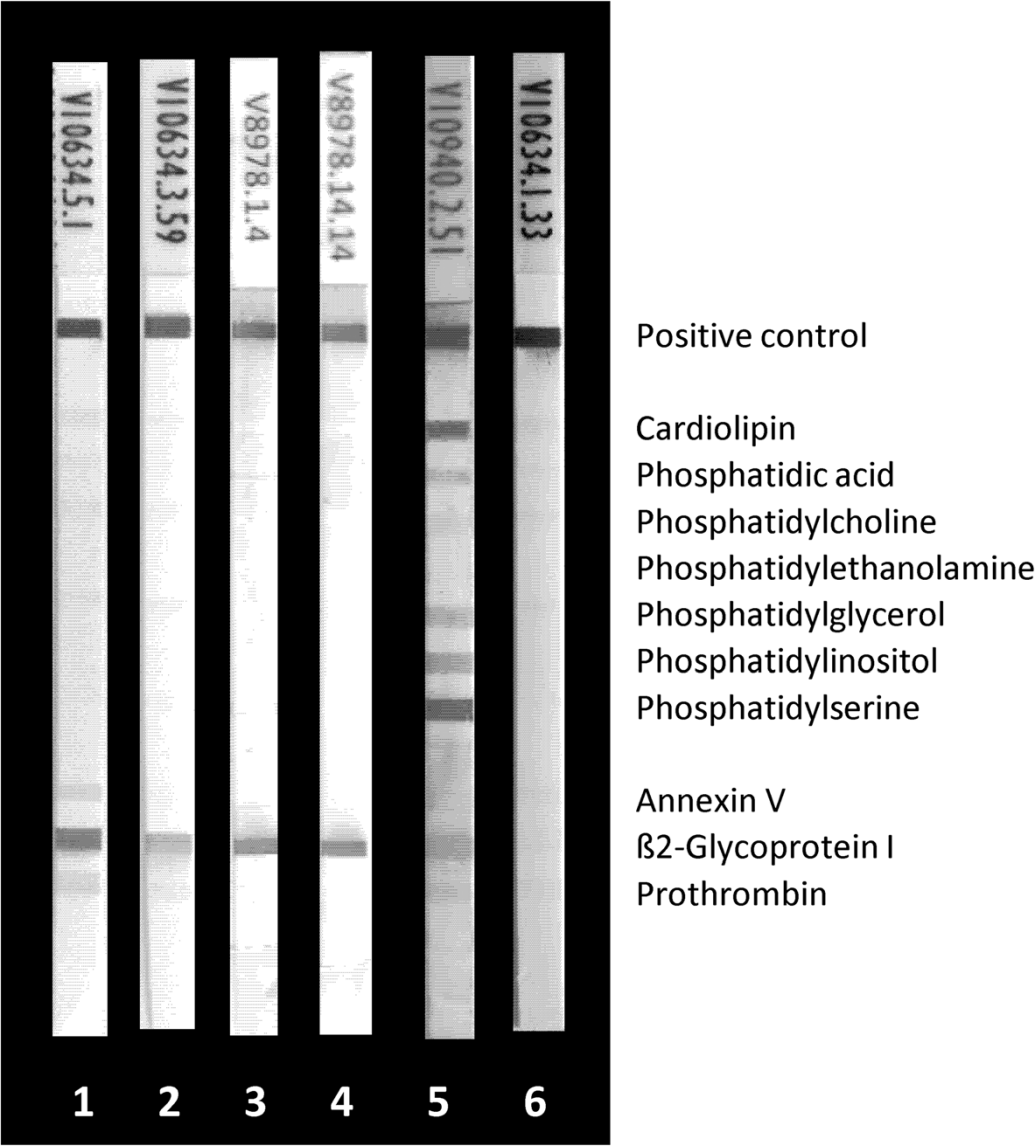
Inhibition of anti-beta2 glycoprotein I (aß2GPI) human monoclonal antibodies (humoAb) MBB2 and HCAL and antiphospholipid (aPL) humoAb RR7F by cardiolipin (CL) micelles in the line immunoassay (LIA): aß2GPI and aPL humoAb were run in the LIA alone or together with 10 mg/L CL micelles. As the positive reaction control a mixture of human IgG and IgM was immobilized. *1*, 0.1 mg/L aß2GPI MBB2; *2*, 0.1 mg/L aß2GPI MBB2 + CL micelles; *3*, 0.02 mg/L aß2GPI HCAL; *4*, 0.02 mg/L aß2GPI HCAL + CL micelles; *5*, 10.0 mg/L aPL RR7F; and *6*, 10.0 mg/L aPL RR7F + CL micelles

The human monoclonal aPL RR7F (IgG) known to be reactive with several PL in a manner completely independent of PL-binding proteins [25] was employed to investigate the reactivity with PL on LIA strips (Fig. 3). It showed reactivity with CL, PA, and PS only. Co-incubation of ß2GPI did not interfere with such a reactivity pattern, whereas serum co-incubation revealed less staining of all bands, probably due to unspecific blocking by serum components (Fig. 3). Interestingly, the reactivity of RR7F to PL was completely blocked by 10 mg/L CL micelles (Fig. 4). In contrast, the aß2GPI reactivity of MBB2 and HCAL was not affected by co-incubation with CL micelles (Fig. 4).

### Comparison of aPL testing by LIA and ELISA

To detect aPL profiles and to analyze possible differences in aPL detection, we tested sera from APS patients and controls by classical ELISA and novel LIA. Comparison of the LIA technique with the aPL assays recommended by the international classification criteria revealed good agreement for IgG/IgM aß2GPI and aCL (Cohen’s *kappa* = 0.78, 95 % CI 0.64, 0.88 and 0.72, 95 % CI 0.62, 0.82, respectively). On the other hand, comparing the study cohorts, the only significant difference was for IgG aCL detected by LIA versus ELISA in asymptomatic aPL+ carriers (McNemar’s test, 29.17 %, 95 % CI 5.27, 29.17, *p* = 0.0156). In contrast, IgM aCL did not demonstrate a significant difference by either method in this group (12.00 %, 95 % CI –8.66, 19.8, *p* = 0.3750). This finding was due to the significantly higher prevalence of IgG/M aCL positive samples detected by ELISA compared with LIA in this group (17/24 vs. 9/24, *p* = 0.0415).

**Fig. 5:**
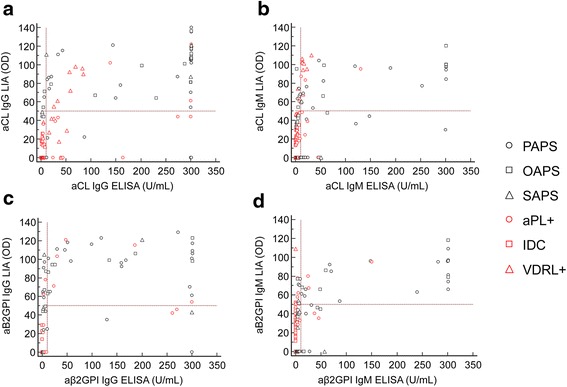
Spearman’s rank correlation analysis of anti-cardiolipin (aCL) IgG (**a**), aCL IgM (**b**), anti-beta 2 glycoprotein I (aß2GPI) IgG (**c**), and aß2GPI IgM (**d**) quantitative testing. Antiphospholipid antibodies were determined in 61 patients with antiphospholipid syndrome (APS) and 146 controls by line immunoassay (LIA) and enzyme-linked immunosorbent assay (ELISA). The following correlation coefficients (ρ) were obtained for aCL IgG and IgM, and aß2GPI IgG and IgM quantitative testing by LIA and ELISA (aCL IgG: ρ = 0.737 95 % CI 0.657, 0.801; aCL IgM: ρ = 0.593 95 % CI 0.482, 0.686; aß2GPI IgG: ρ = 0.774 95 % CI 0.702, 0.830; aß2GPI IgM: ρ = 0.566 95 % CI 0.449, 0.663, respectively; p < 0.001). *aPL+* asymptomatic patients with autoantibodies to phospholipids, *IDC* infectious diseases controls, *OAPS* obstetric antiphospholipid syndrome, *PAPS* primary antiphospholipid syndrome, *SAPS* secondary antiphospholipid syndrome, *VDRL+* Venereal Disease Research Laboratory test positive, *OD* optical density

Comparing the distinct isotypes of the recommended aPL detected by LIA and ELISA in 161 patients with APS and 156 controls, there was good agreement for aCL and aß2GPI IgG (Cohen’s *kappa* = 0.75 and 0.78, respectively) as well as for aCL and aß2GPI IgM (Cohen’s *kappa* = 0.64 and 0.65, respectively; Additional file 2: Table S2). On quantitative analysis of aCL IgG and IgM, and aß2GPI IgG and IgM, by LIA and ELISA there was significant correlation, with Spearman correlation coefficients (ρ) ranging from 0.566 to 0.774, respectively (*p* < 0.001), (Fig. 5).

### Comparison of aPL testing in APS patients and controls

**Fig. 6:**
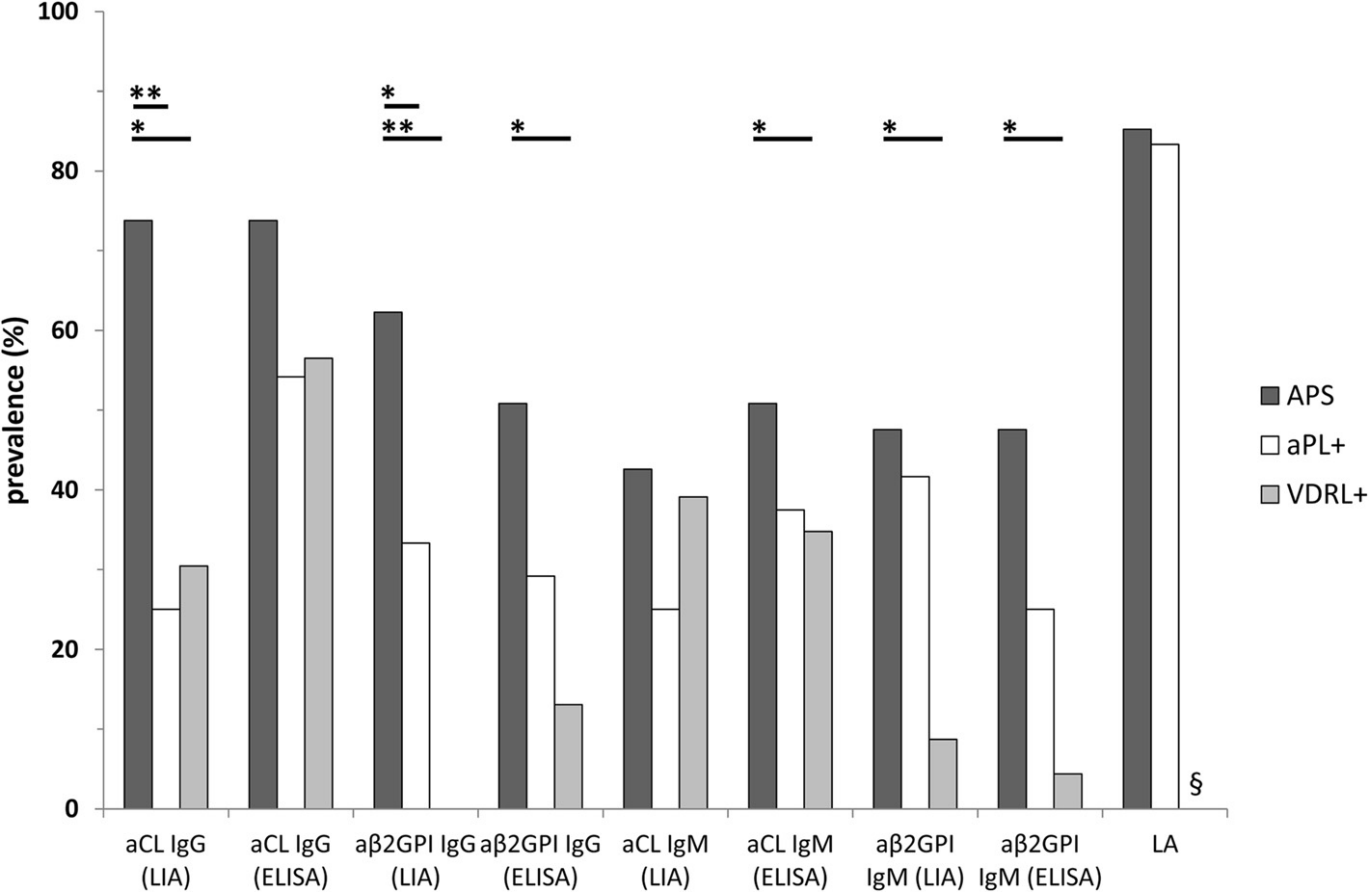
Prevalence of consensus criteria antiphospholipid antibodies (aPL) tested by line immunoassay (LIA), enzyme-linked immunosorbent assay (ELISA), and lupus anticoagulant (LA) analysis in 61 patients with antiphospholipid syndrome (APS), 24 aPL-positive (aPL+) individuals, and 23 Veneral Disease Laboratory test-positive (VDRL+) patients. *aß2GPI* antibeta2-glycoprotein I, *aCL* anticardiolipin, *aPA* antiphosphatidic acid, *aPE* antiphosphatidylethanolamine, *aPG* antiphosphatidylglycerol, *aPI* antiphosphatidylinositol, *aPL+* asymptomatic carriers with autoantibodies to phospholipids, *aPS* antiphosphatidylserine. *P < 0.05; **p < 0.0001; ^§^not done

As expected, patients suffering from APS (*n* = 61) had significantly higher prevalence of IgG/IgM aCL and aß2GPI detected by ELISA compared with those in IDC and HS (Table 2). However, comparing APS with VDRL+ patients, only IgG/IgM aß2GPI were significantly more prevalent in patients with APS (Fig. 6). In contrast, in asymptomatic aPL+ carriers the prevalence of all aPL detected by ELISA was not significantly different compared with patients suffering from full-blown APS (Fig. 6).

**Table 2:** Antiphospholipid antibody (aPL) positive sera tested by enzyme-linked immunosorbent immunoassay (ELISA) and line immunoassay (LIA) in 61 patients with antiphospholipid syndrome (APS) and 146 controls

ELISA						LIA																					LA
	aCL		aß2GPI		Any aPL	aCL		aPA		aPC		aPE		aPG		aPI		aPS		aAnV		aß2GPI		aPT		any aPL	
	G	M	G	M	G/M	G	M	G	M	G	M	G	M	G	M	G	M	G	M	G	M	G	M	G	M	G/M	
APS *n* = 61	45	31	31	29	46	45	26	31	25	0	0	0	11	18	12	25	16	43	28	3	4	38	29	8	11	53	52
PAPS *n* = 34	31	21	21	20	31	29	18	20	18	0	0	0	7	13	10	17	11	29	20	1	3	25	19	6	6	31	29
PAPS/T *n* = 29	27	18	17	17	27	25	15	17	15	0	0	0	4	11	9	13	10	25	17	1	2	21	16	5	4	26	24
PAPS/TO *n* = 5	4	3	4	3	4	4	3	3	3	0	0	0	3	2	1	4	1	4	3	0	1	4	3	1	2	5	5
OAPS *n* = 22	11^§^	8	8	8	12^§^	13^§^	7	8	6	0	0	0	4	4	2	7	4	11^§^	7	2	1	10	8	1	4	18	19
SAPS *n* = 5	3	2	2	1	3	3	1	3	1	0	0	0	0	1	0	1	1	3	1	0	0	3	2	1	1	4	4
aPL + *n* = 24	13	9	7	6	17	6**	6	6***	6	0	0	0	0*	3	1	4*	3	5**	6	0	2	8*	10	1	1	17	20
IDC *n* = 50	3**	1**	0**	0**	4**	0**	0**	0**	0**	0	0	0	0*	0**	0*	0**	1*	0**	0**	2	10*	0**	1**	1*	4	16**	nd
VDRL + *n* = 23	13	8	3*	1*	15	7*	9	0**	1*	0	0	0	1	0*	1*	0*	1*	0**	1*	1	2	0**	2*	0	5	12*	nd
HS *n* = 49	0**	2**	0**	0**	2**	1**	1**	1**	0**	0	0	0	4	0**	0*	1**	0**	1**	0**	0	0	0**	0**	0*	0*	6**	nd

*P < 0.05 and **p < 0.0001 for comparison of prevalence of aPL in patients with APS (*n* = 61) and the respective control cohort; ***trend towards difference in prevalence of aPL in patients with APS (*n* = 61) and the respective control cohort (*p* = 0.05). ^§^ *P* < 0.05 for comparison of prevalence of aPL in PAPS/T patients (*n* = 29) with SAPS or OAPS patients. *aAnV* antiannexin V, *aß2GPI* antibeta2-glycoprotein I, *aCL* anticardiolipin, *aPA* antiphosphatidic acid, *aPC* antiphosphatidylcholine, *aPE* antiphosphatidylethanolamine, *aPG* antiphosphatidylglycerol, *aPI* antiphosphatidylinositol, *aPL+* asymptomatic patients with autoantibodies to phospholipids, *aPS* antiphosphatidylserine, *aPT* antiprothrombin, *HS* healthy subjects, *IDC* infectious diseases controls, *LA* lupus anticoagulant, *nd* not determined, *OAPS* obstetric primary antiphospholipid syndrome, *PAPS* primary antiphospholipid syndrome, *PAPST* primary antiphospholipid syndrome with thrombotic events, *PAPS/TO* primary antiphospholipid syndrome with thrombotic and obstetric manifestations, *SAPS* secondary antiphospholipid syndrome, *VDRL+* Venereal Disease Research Laboratory test-positive

As a matter of fact, IgG/IgM reactivity against CL and ß2GPI by LIA was significantly higher in APS samples vs. HS and IDC groups as detected by ELISA. Of note, IgG binding to CL and ß2GPI in the LIA was significantly lower in aPL+ carriers and VDRL+ samples than in APS (Fig. 6). Sera that were positive in aCL and/or aß2GPI ELISAs also displayed reactivity with the anionic PL in the LIA strips. Only a minority of samples displayed reactivity against PT, or AnV and PE (mainly of IgM isotype) (Table 2).

**Fig. 7:**
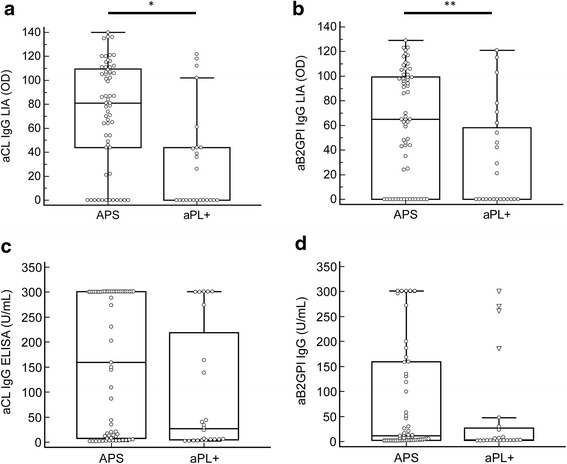
Comparison of quantitative anti-cardiolipin (aCL) IgG (**a**) and anti-beta 2 glycoprotein I (aß2GPI) IgG (**b**) analysis by line immunoassay (LIA) and enzyme-linked immunosorbent assay (ELISA) (**c**, **d**, respectively) in patients with antiphospholipid syndrome (APS) (*n* = 61) and antiphospholipid antibody-positive carriers (aPL+) (*n* = 24). In the box and whisker plots, outlier values are defined as values that are lower than the lower quartile minus three times the interquartile range, or higher than the upper quartile plus three times the interquartile range, displayed as *triangles*. *P = 0.0005; **p = 0.0066

The significant reduced prevalence of aCL and aß2GPI IgG analyzed by LIA in aPL+ carriers compared to APS patients were confirmed by quantitative LIA testing (Fig. 7). The median optical density (OD) level of aCL IgG in aPL+ carriers (0.1, 95 % CI 0.1, 43.3) was significantly lower than in APS patients (81.0, 95 % CI 65.8, 101.6, *p* = 0.0005). Likewise, the median OD level of aß2GPI IgG in aPL+ carriers (0.1, 95 % CI 0.1, 48.0) was significantly reduced in contrast to the one in APS patients (65.0, 95 % CI 46.8, 93.2, *p* = 0.0066). Such significant differences were not observed by quantitative aCL and aß2GPI IgG ELISA analysis (*p* > 0.05, respectively) (Fig. 7).

In order to investigate the reasons for significant differences on qualitative and quantitative LIA testing of APS patients and controls, we tested nine patients with APS and aPL IgG reactivity to D1 of ß2GPI only and five with aPL reactivity to D4/5 only. Domain reactivity was determined by research assays as described in “Methods”. Remarkably, nine of nine patients with APS and sole D1 reactivity scored positive in the LIA, whereas, in contrast none of the five patients with APS and D4/5 reactivity were positive (Table 3) (*p* = 0.0005).

**Table 3:** Line immunoassay (LIA) reactivity of antibeta2-glycoprotein I (aβ2GPI) domain 1 (D1) negative-D4/5 positive and D1 positive-D4/5 negative sera from patients (Pts) with antiphospholipid syndrome (APS)

		aPL IgG reactivity by LIA (OD)									
aβ2GPI reactivity profile	Pt #	aCL	aPA	aPC	aPE	aPG	aPI	aPS	aAnV	aβ2GPI	aPT
aD1 negative-aD4/5 positive	1	0	0	0	0	0	0	0	0	20	0
	2	0	0	0	0	0	0	0	0	30	0
	3	0	17	0	0	0	0	0	0	0	0
	4	0	0	0	0	0	0	0	0	27	0
	5	0	0	0	0	0	0	0	0	34	0
aD1 positive-aD4/5 negative*	6	*108*	*80*	0	0	0	0	*109*	0	*97*	0
	7	*121*	*107*	0	35	*84*	*94*	*116*	44	*121*	*83*
	8	*87*	45	0	0	41	*58*	*92*	0	*65*	0
	9	*111*	*68*	0	0	*85*	*91*	*101*	0	*98*	37
	10	*136*	*107*	0	0	*109*	*117*	*131*	33	*123*	*80*
	11	*99*	*66*	0	0	49	*69*	*93*	0	*86*	0
	12	*136*	*104*	0	0	*99*	*94*	*121*	*56*	*123*	*82*
	13	*116*	*83*	0	0	*100*	*106*	*126*	0	*118*	*33*
	14	*135*	*121*	0	0	*91*	*106*	*121*	0	*116*	*50*

Results of antiphospholipid (aPL) IgG are expressed as optical density (OD) values whereas OD values equaling or above 50 were scored positive (positive values in italics). *aAnV* antiannexin V, *aCL* anticardiolipin,*aPA*, antiphosphatidic acid; *aPC*, antiphosphatidylcholine; *aPE* antiphosphatidylethanolamine, *aPG* antiphosphatidylglycerol, *aPI* antiphosphatidylinositol, *aPS* antiphosphatidylserine, *aPT* antiprothrombin. *P = 0.0005 vs aD1 negative-aD4/5 positive

Notably, LA analysis, like ELISA testing, did not reveal significant differences in the prevalence of aPL when comparing patients with APS to asymptomatic aPL+ carriers. Altogether, only aPL IgG analysis by LIA discriminated APS patients from aPL+ carriers.

In this study, evaluating at least one positive assay, the multiplex LIA demonstrated sensitivity of 86.9 % compared to 75.4 % by single ELISAs and 85.2 % by LA analysis. By employing different disease controls, the specificity reached similar values of 74 % for ELISA and 72 % for LIA testing when excluding aAnV IgM, which was positive in all patients with CMV infection (*n* = 10). Sensitivity of 67.2 % by LIA vs. 58.8 % by ELISA at a higher specificity of around 95 % for both techniques has been reported in an earlier study (Table 4).

**Table 4:** Comparison of the performance characteristics of enzyme-linked immunosorbent assay (ELISA), line immunoassay (LIA) and lupus anticoagulant (LA) testing of this study investigating 61 patients with APS and 156 controls with previously published data comprising 85 patients with APS and 144 controls [17]

Cohorts		Sensitivity	95 % CI	Specificity	95 % CI	+LR	95 % CI	-LR	95 % CI
61 Patients with APS, 157 controls including 25 asymptomatic carriers	ELISA	75.4	62.7–85.5	74.0	66.1–80.9	2.9	2.1–4.0	0.3	0.2–0.5
	LIA*	86.9	75.8–94.2	78.8	71.2–85.1	4.1	3.0–5.7	0.2	0.1–0.3
85 Patients with APS, 144 controls [17]	ELISA	58.8	47.6–69.4	95.8	91.1–98.5	14.1	6.3–31.5	0.4	0.3–0.6
	LIA	67.1	56.0–76.9	96.5	92.1–98.9	19.3	8.1–46.3	0.3	0.2–0.5

*Anti-annexin V antibody data for patients with cytomegalovirus infection (*n* = 10) were excluded. *CI* confidence interval, *LR* likelihood ratio

### Comparison of aPL testing in APS patient subgroups

Comparing patients suffering from PAPS/T and OAPS, there was significantly higher prevalence of aCL determined by ELISA in patients with PAPS/T (27/29 vs. 11/22, *p* = 0.0001). Further, the prevalence of at least one aPL positivity detected by ELISA (aß2GPI IgG/IgM and/or aCL IgG/IgM positive) was also significantly higher in PAPS/T patients compared to those with OAPS (27/29 vs. 12/22, *p* = 0.0021). aCL and aPS IgG were also significantly more prevalent in patients with PAPS/T compared to those with OAPS (*p* = 0.04973 and *p* = 0.01154, respectively) in the LIA test. Of note, there was no significant difference in the prevalence of aPL when comparing patients with PAPS/T and OAPS in analysis of LA.

## Discussion

The persistent presence of aPL is the serological hallmark of APS and represents one of its mandatory classification criteria [2]. It is a well-accepted consensus that aPL interact with PL-binding proteins. While most of the reactivity was against β2GPI, additional PL-binding proteins were shown to be recognized by aPL [11–13, 16]. The different aPL subpopulations cannot be detected by a single diagnostic assay and this supports the recommendation for performing aCL, aβ2GPI, and LA assays in order to identify all the potential aPL.

Between 1 % and 5 % of healthy individuals have circulating aPL that are detectable with the currently recommended aPL assays [4]. This raises the issue of identifying the truly diagnostic aPL and/or those aPL that are really predictive for the clinical manifestations of the syndrome.

The LIA membrane strips provide a unique matrix that allows PL to mimic their natural conformation in tissues as reported for other amphiphatic non-protein antigenic molecules [28, 31–33]. Hence, immobilized PL offer a suitable binding substrate for the main PL-binding proteins. Accordingly, this study investigated the performance of a novel LIA hydrophobic solid phase for the simultaneous detection of multiple aPL in a well-defined cohort of patients with APS and controls including aPL+ asymptomatic carriers.

Of note, the novel LIA solid phase has already proven its usefulness for the specific analysis of auto-antibodies to lipopolysaccharides and glycolipids exhibiting PL-like physicochemical characteristics [28, 33]. In contrast to the planar ELISA solid phase, the porous hydrophobic LIA membrane is assumed to incorporate the hydrophobic PL tail. This shields the by far larger tail of the amphiphatic PL molecule from the reaction environment and, thus, prevents unspecific interactions [11]. Of note, the humoAb RR-7 F interacting with anionic PL only in ELISA also bound readily immobilized anionic PL in the investigated LIA [25]. This reactivity was completely inhibited by CL micelles that expose only hydrophilic CL-heads on their surface in aqueous solutions. Consequently, this confirms the interaction of RR-7 F with the hydrophilic PL-heads on the PVDF membrane. The aβ2GPI humoAbs MBB2 and HCAL were able to recognize their target molecule coated on the membrane as well as to react with anionic PL through the bound cationic β2GPI.

Interestingly, the addition of serum or purified β2GPI to MBB2 revealed different binding characteristics to the immobilized anionic PL in the novel reaction environment. CL, also referred to as diphosphatidylglycerol, binds β2GPI far better than its monomeric variant PG. Otherwise, PS bearing only one phosphatic group has a better binding than PI or PG. This supports the assumption that the number, orientation, and accessibility of anionic phosphatic groups in the hydrophilic PL heads determine the binding of β2GPI and consequently of the β2GPI-dependent aPL. As MBB2 has been demonstrated to specifically react with D1 of ß2GPI and its conformational epitope [24], its binding to the LIA strips indicates that the immobilized β2GPI readily exposes D1. This demonstrates the accessibility of this important pathogenic epitope-bearing domain in the LIA reaction environment. As a consequence, these variables may affect the ultimate serum autoantibody binding.

Indeed, favorable assay performance particularly for the specificity of this novel LIA technique for the analysis of aPL has been reported recently and was confirmed in this study [17, 21]. In fact, the prevalence of aCL and aß2GPI IgG was significantly reduced in aPL+ carriers and in VDRL+ individuals compared to patients with APS when analyzed by LIA but not by ELISA. Of note, the significantly qualitative differences of aCL IgG and aß2GPI IgG testing by LIA in APS patients and aPL+ carriers were confirmed by quantitative analyses of the respective median OD levels.

We could not determine the binding of either serum or purified PT with immobilized anionic PL, particularly with PS, in the multiplex LIA environment as shown for β2GPI. Even in the presence of Ca^+2^ ions, which are required for the aPT/PS ELISA reaction environment, no binding was detected. However, it cannot be excluded that the PT in the LIA reaction environment is not able to change its conformation in the presence of Ca^2+^ ions. This point could not be addressed by our experimental setting. Addition of further PT in the presence of the right high Ca++ concentration did not change the binding behavior. Of note, there was a significantly different prevalence of aPT and aPS IgG/IgM (*p* < 0.0001 and *p* = 0.0017, respectively) in APS patients by LIA. Altogether, this indicates that aPS reactivity in LIA is mainly based on the reactivity to β2GPI bound to PS and does not involve PT present in the serum sample. Theoretically in fact, serum PT could bind PS on the PVDF membrane. Accordingly, in our study there was no significant correlation between thrombotic events and either aPT or aPS detected by LIA, which was reported for aPS/PT elsewhere [12]. The latter findings is in line with the hypothesis that aPS/PT abs recognize a peculiar epitope(s) exposed on PT only when complexed with PS-coated polystyrene plates.

A further novelty of this aPL assay is the possibility of detecting antibodies against D1 because of the way the molecule is oriented on the matrix [34, 35]. After binding of β2GPI to negatively charged surfaces like immobilized anionic PL by D5 (containing the PL-binding site), D1 forms the top of the induced fishhook-like β2GPI structure that is predisposed to interact with aPL [35]. Assuming a high density of the hydrophilic PL heads on the LIA membrane, β2GPI D4 and D5 could be indeed engaged in the binding of immobilized PL and no longer available for aPL interaction. Indeed, none out of five patients with APS with D4/D5 reactivity only, had a positive aPL IgG by LIA, whereas all nine patients with D1 reactivity only did have positive aPL by LIA. aPL directed against D1 are significantly more present in sera in APS than in pathological controls such as infectious patients or aPL+ asymptomatic carriers. On the other hand, aPL reacting with β2GPI D4 and D5 display opposite behavior [36, 37]. Indeed, there is a significant difference in the analysis of aPL binding to anionic PL/β2GPI complexes in LIA compared with ELISA in VDRL+ patients and in particular in asymptomatic aPL+ carriers. Thus, the significantly lower prevalence of aPL detected by LIA in aPL+ carriers and in VDRL+ patients indicates more specific detection of diagnostic aPL by LIA. Remarkably, on aPL analysis by LA testing and ELISA there were no such significant differences, which suggests the detection of aPL to epitopes other than those present on D1. As the aPL reactivity to D4-5 has been reported not to be associated with thromboembolism, our data further support the suggestion that LIA may detect aPL that are more predictive of clinical events in APS [36–38].

## Conclusions

This is the first study reporting on a diagnostic assay for discrimination of patients with APS from asymptomatic aPL carriers and patients with infectious diseases. The varying aPL reactivity detected in LIA could be explained by conformational differences in the fishhook-like structure of β2GPI interacting with PL heads homogenously oriented on the membrane.

## Additional files

Additional file 1: Table S1. Clinical and laboratory features of patients with antibeta2-glycoprotein I (aβ2GPI) domain 1 (D1) antibody negative-D4/5 positive and D1 positive-D4/5 negative antiphospholipid syndrome (APS). (DOCX 23 kb) Additional file 2: Table S2. Comparison of antiphospholipid antibody (aPL) testing by line immunoassay (LIA) and enzyme-linked immunosorbent assay (ELISA) in 61 patients with APS and 156 controls. (DOCX 16 kb)

## Abbreviations

aAnV: antiannexin V antibody
aCL: anti-cardiolipin antibody
AnV: annexin V
aPL: antiphospholipid antibody(ies)
aPL+: antiphospholipid antibody positive
APS: antiphospholipid syndrome
aPT: antiprothormbin antibody
BSA: bovine serum albumin
CL: cardiolipin
D1-D5: domains 1-5
ELISA: enzyme-linked immunosorbent assay
HS: healthy subjects
humoAb(s): human monoclonal antibody(ies)
IDC: infectious diseases controls
LA: lupus anticoagulant
LIA: line immunoassay
mAb: monoclonal antibodies
OAPS: obstetric primary antiphospholipid syndrome
OD: optical density
PAPS: primary antiphospholipid syndrome
PBS: phosphate-buffered saline
PC: phosphatidylcholine
PE: phosphatidylethanolamine
PG: phosphatidylglycerol
PI: phosphatidylinositol
PL: phospholipid
PS: phosphatidylserine
PT: prothrombin
PVDF: polyvinylidene difluoride
SAPS: secondary antiphospholipid syndrome
VDRL+: Venereal Disease Research Laboratory test positive
β2GPI: beta2-glycoprotein I
