# Supplementary material for: Antiphospholipid antibodies detected by line immunoassay differentiate among patients with antiphospholipid syndrome, with infections and asymptomatic carriers
Source: Arthritis Res Ther. 2016 May 21;18:111. doi: 10.1186/s13075-016-1018-x (PMC4875598; doi:10.1186/s13075-016-1018-x)
Supplement: Additional file 1: Table S1. — Clinical and laboratory features of patients with antibeta2-glycoprotein I (aβ2GPI) domain 1 (D1) antibody negative-D4/5 positive and D1 positive-D4/5 negative antiphospholipid syndrome (APS). (DOCX 23 kb) [file 13075_2016_1018_MOESM1_ESM.docx]

Supplementary Table 1: Clinical and laboratory features of antibeta2-glycoprotein I (aβ2GPI) domain 1 (D1) antibody-negative – D4/5 positive and D1 positive – D4/5 negative antiphospholipid syndrome (APS) patients

| *Pt # | Sex | Diagnosis | AT | VT | EP | IUD | PB | IUGR | Preecl/Ecl | LA | aCL-M | aCL-G | aβ_2_GPI-M | aβ_2_GPI-G | aβ2GPI-D1 | aβ2GPI-D4/5 |
| --- | --- | --- | --- | --- | --- | --- | --- | --- | --- | --- | --- | --- | --- | --- | --- | --- |
| 1 | F | OAPS | 0 | 0 | 0 | 0 | 1 | 1 | 1 | 0 | 1.00 | 2.00 | 0.00 | 48.30 | 19.72 | *57.70* |
| 2 | F | OAPS | 0 | 0 | 0 | 1 | 0 | 0 | 0 | 0 | 2.00 | 4.00 | 6.45 | 63.00 | 13.20 | *64.80* |
| 3 | M | PAPS/T | 1 | 0 | na | na | na | na | na | 0 | 9.00 | 2.00 | 4.52 | 67.00 | 6.30 | *40.70* |
| 4 | F | aPL + | 0 | 0 | 0 | 0 | 0 | 0 | 0 | 0 | 8.00 | 4.00 | 12.25 | 53.20 | 13.30 | *50.00* |
| 5 | F | aPL + | 0 | 0 | 0 | 0 | 0 | 0 | 0 | 0 | 2.00 | 2.00 | 5.16 | 70.00 | 8.00 | *134.00* |
|  |  |  |  |  |  |  |  |  |  |  |  |  |  |  |  |  |
| 6 | F | PAPS/T | 0 | 1 | 0 | 0 | 0 | 0 | 0 | 1 | 3.00 | 77.00 | 5.16 | 59.60 | *55.40* | 6.30 |
| 7 | F | PAPS/T | 0 | 1 | 0 | 0 | 0 | 0 | 0 | na | 0.00 | 50.00 | 0.00 | 77.30 | *121.00* | 13.90 |
| 8 | F | PAPS/TO | 1 | 0 | 1 | 0 | 0 | 0 | 0 | 1 | 7.00 | 112.00 | 6.45 | 38.70 | *31.70* | 10.60 |
| 9 | F | OAPS | 0 | 0 | 1 | 0 | 0 | 1 | 0 | 1 | 24.00 | 88.00 | 18.71 | 91.70 | *112.30* | 7.70 |
| 10 | M | PAPS/T | 1 | 1 | na | na | na | na | na | 1 | 10.00 | 131.00 | 18.00 | 73.00 | *59.90* | 8.00 |
| 11 | F | OAPS | 0 | 0 | 0 | 0 | 1 | 1 | 1 | 1 | 11.00 | 103.00 | 10.32 | 29.60 | *32.50* | 7.50 |
| 12 | F | OAPS | 0 | 0 | 0 | 1 | 0 | 0 | 0 | 1 | 17.00 | 86.00 | 36.77 | 84.30 | *145.40* | 8.40 |
| 13 | M | PAPS/T | 0 | 1 | na | na | na | na | na | 1 | 18.00 | 176.00 | 29.00 | 58.70 | *41.50* | 11.80 |
| 14 | F | PAPS/T | 1 | 0 | 0 | 0 | 0 | 0 | 0 | 1 | 2.00 | 88.00 | 13.55 | 77.00 | *68.50* | 9.80 |

*Pt # 1-5, aβ2GPI D1 negative - D 4/5 positive patients; Pt # 6-14, aβ_2_GPI D1 positive - D4/5 negative patients.

Anti-cardiolipin antibody (aCL) and aβ2GPI values are expressed as U/ml; cut-off: 10 U/ml aCL and aβ_2_GPI, 20 U/ml aβ2GPI D1 and aβ2GPI D4/5.

OAPS, obstetric primary antiphospholipid syndrome; PAPS/T, primary antiphospholipid syndrome with thrombotic events; PAPS/TO, primary antiphospholipid syndrome with thrombotic and obstetric manifestations; aPL+, asymptomatic patients with autoantibodies to phospholipids; AT, arterial thrombosis; VT, venous thrombosis; EP, early pregnancy loss; IUD, intrauterine death; PB, premature birth; IgG, G; IgM, M; IUGR, intrauterine growth retardation; Preecl/Ecl, preeclampsia/eclampsia; LA, lupus anticoagulant; aβ2GPI-D1, anti-β2GPI Domain 1 IgG antibodies; aβ2GPI-D4/5, anti-β2GPI Domain 4/5 IgG antibodies; 0, absent; 1, present; na, not applicable.
