# Supplementary material for: Antiphospholipid antibodies detected by line immunoassay differentiate among patients with antiphospholipid syndrome, with infections and asymptomatic carriers
Source: Arthritis Res Ther. 2016 May 21;18:111. doi: 10.1186/s13075-016-1018-x (PMC4875598; doi:10.1186/s13075-016-1018-x)
Supplement: Additional file 2: Table S2. — Comparison of antiphospholipid antibody (aPL) testing by line immunoassay (LIA) and enzyme-linked immunosorbent assay (ELISA) in 61 patients with APS and 156 controls. (DOCX 16 kb) [file 13075_2016_1018_MOESM2_ESM.docx]

Supplementary Table 2: Comparison of antiphospholipid antibody (aPL) testing by line immunoassay (LIA) and enzyme-linked immunosorbent assay (ELISA) in 61 patients with APS and 156 controls.

| **ELISA** | | **LIA** | | **Cohen’s kappa** | **95% CI** |
| --- | --- | --- | --- | --- | --- |
| n = 207 | | pos | neg |  |  |
| aCL IgG | pos | 55 | 19 | 0.75 | 0.66 – 0.85 |
|  | neg | 4 | 139 |  |  |
| aß2GPI IgG | pos | 36 | 5 | 0.78 | 0.68 – 0.89 |
|  | neg | 10 | 156 |  |  |
| aCL IgM | pos | 34 | 18 | 0.64 | 0.52 – 0.77 |
|  | neg | 8 | 147 |  |  |
| aß2GPI IgM | pos | 28 | 8 | 0.65 | 0.52 – 0.79 |
|  | neg | 14 | 157 |  |  |

aß2GPI, antibeta2-glycoprotein I; aCL, anticardiolipin
